# Psychology in the Post-Truth Era

**DOI:** 10.5964/ejop.v13i3.1509

**Published:** 2017-08-31

**Authors:** Vlad Petre Glăveanu

**Affiliations:** a Webster University Geneva, Geneva, Switzerland

It is always difficult to define a historical period while being part of it; such work of classification is usually done afterwards with the benefit of hindsight. And yet, following the rise of nationalism and xenophobia in the West, and other places around the world, aided by the spread of lies through populist propaganda, some distinctive ways of referring to current events emerged. ‘Post-truth’ is chief among them. In fact, the notion of ‘post-truth politics’ even entered the dictionaries recently, a term used to define a period in which “objective facts are less influential in shaping public opinion than appeals to emotion and personal belief”^i^. The existence of propaganda and its mission to conquer ‘hearts’ over ‘heads’ is not new here. Neither is the fact that public opinion is shaped by emotions and personal meaning-making. If anything, it would be hard to be otherwise. What is particularly important – and particularly troubling – about this new era is the dismissal of or disregard for “objective facts”. And, more than this, the fact that we live in a completely different informational and technological landscape than a few decades ago. The use of the Internet and social media as main sources of information and arenas of socialisation had consequences few could foresee. The great hopes of the founders of such projects were that they would serve as spaces of free dialogue and exchange, increase communication and, with it, mutual respect and tolerance. The reverse has often been the case. ‘Social media bubbles’ in which ‘alternative facts’, including alternative histories, circulate as true, are of concern today for policy makers, media experts, educators and psychologists alike.

The latter, I argue, have a fundamental contribution to make in the current context. It is not only the case that psychology – at least its social, cultural, cognitive and political branches – deals with public opinion and the emergence and transformation of (social) knowledge. What matters most is that psychology cannot stop at simply discovering new biases and errors in thinking processes, new dynamics within inter-group relations, or listing new forms of manipulation. It is imperative, now more than ever, that psychologists use their expertise to devise practical tools for cultivating critical thinking and reflexivity in relation to a number of areas of social life – e.g., history, politics, economics – and in relation to the distinction between personal beliefs and objective facts. This is not to say that the former are not to be trusted or should be eradicated in the quest for “perfect objectivity”. Far from it, psychology’s key role in this post-truth era should be to help people distinguish between beliefs and facts and understand the strengths and limitations associated with each. This is all the more important in relation to social media and in view of the kind of informational literacy that should be developed in new (and old) generations of users. Being able to appreciate the soundness of the information found on social media is a crucial skill that needs to be educated from early on. Unlike a legal or political approach, that might focus on criminalising the spreading of falsehoods on the Internet, psychologists should be concerned with studying and developing the meta-cognitive skills required to search for, evaluate and make use of information in online or offline environment. It is this kind of literacy that needs both increased research attention and the efforts of practitioners to translate theory into concrete tools and motivate people to make good use of them.

It is a well-known fact that psychology is one of the first disciplines to be shut down under authoritarian or totalitarian regimes. More than this, the accusations usually brought against psychologists are those of intellectualism and of trying to undermine the state (typically the single or dominant party). One of the worrying signs of this dynamic in the ‘post-truth era’ takes the form of contempt for experts and expertise. Psychological expertise is often disregarded on the basis that it serves a particular agenda and that it misconstrues human behaviour. While a close scrutiny of the validity of research is certainly needed and the discipline itself has in place a series of institutional practices dedicated to this exercise, we must resist as a community the temptation of isolating ourselves to the ivory tower of science and failing to reach out to the various groups of people we ultimately aim to understand and to help. In addition, we need to find new ways to disseminate psychological information, to increase awareness about its utility and its multiple practical applications. In the end, it is by demonstrating the value of one’s research (whether psychology or multidisciplinary) that we can hope to make an impact outside of the academic world and of our own ‘bubble’ populated by colleagues who hold similar beliefs and values. This is not a call to turn psychologists into activists but rather one encouraging colleagues to reflect more on the practical implications of their research – whatever field of psychology it belongs to – and the ways in which they contribute, through their work, to current debates.

What is at stake in defining a new role for psychology within the post-truth era is not only the future of the discipline, faced with the double challenge of anti-intellectualism and increased authoritarianism, but also the way we understand and foster democratic values and civic participation. A psychological science aimed at strengthening the foundations of open and tolerant societies, based on ideals of social justice and embracing diversity is essential for the development of societies that are resilient to nationalism, populism, and discriminatory practices. This effort needs to be reflected in the quality of the work done by psychologists as well as its wider dissemination, including through making it more accessible for a general public. In this context, the development and growth of open access sources of credible, high-quality psychological information plays a special part. Europe’s Journal of Psychology (EJOP) has been at the forefront of this trend for almost 13 years and we continuously strive to improve our content and to reach wider audiences.

Given the particular challenges associated with living and working in an age of ‘post-truth politics’, we have been and continue to welcome manuscripts – including the proposal of special editions – that address pressing societal issues such as current uses of social media, inequality and social justice, migration and multiculturalism, terrorism, democracy and democratic values, protest and civic participation, climate change and environmental concerns, political behaviour and the psychology of human rights, among others. We are also searching for innovative ways to make psychological information available to non-expert audiences without compromising on the scientific standards of conducting and reporting research. These ways could include the option of writing non-technical abstracts or article summaries for lay people as well as asking authors to include more substantial sections on the implications and practical uses of their research, written with a wider audience in mind.

In the end, the ‘post-truth era’ of today might very well be a momentary, negative development soon overcome and even forgotten. This is a rather optimistic view given the fact that its consequences are already being felt by various communities around the world: refugees unable to find shelter from war, protesters detained for expressing their dissatisfaction, people being displaced or losing their livelihood due to climate change, those who feel powerless in the face of growing inequality or afraid they might be killed or evacuated due to imminent conflicts. These are all part of the realities of today even more so than a decade ago given the increasing scorn for and dismissal of evidence coupled with embracing more or less unfounded views just because they suit one’s interests and reflect one’s emotions. A thorough psychological analysis of these phenomena is timely and necessary, as is the creation of practical tools to counter ‘post-truth’ mentalities.

Our hope at EJOP is to make a positive contribution in this regard by opening the journal even more to critical inquiries about social issues. There is still a possibility that the notion of ‘post-truth’ will be remembered in a few years as a short-lived historical anomaly. For this to be the case, though, psychologists need to play a more active part in their society as scientists, practitioners and, above all else, as agents of change.

